# COVID-19 information and self-protective behaviors among rural communities in tropical forests

**DOI:** 10.1186/s12889-022-13772-y

**Published:** 2022-07-20

**Authors:** Yoshito Takasaki, Oliver T. Coomes, Christian Abizaid

**Affiliations:** 1 Graduate School of Economics, University of Tokyo, Tokyo, Japan; 2 Department of Geography, McGill University, Montreal, QC Canada; 3 Department of Geography & Planning and School of the Environment, University of Toronto, Toronto, ON Canada

**Keywords:** COVID-19 information, Self-protective behaviors, Media, Trust, Rural communities, Amazonia

## Abstract

**Background:**

Health risk communication plays a key role in promoting self-protective measures, which are critical in suppressing COVID-19 contagion. Relatively little is known about the communication channels used by rural poor populations to learn novel measures and their effectiveness in promoting self-protective behaviors. Behavioral change can be shaped by people’s trust in government institutions which may be differentiated by social identity, including indigeneity.

**Methods:**

During an early phase of the pandemic, we conducted two telephone surveys with over 460 communities – both Indigenous and mestizo – without road access and limited communication access in the Peruvian Amazon. This is the first report on the association of information sources about self-protective measures against COVID-19 with the adoption of self-protective behaviors in remote rural areas in developing countries.

**Results:**

People mainly relied on mass media (radio, television, newspapers) and interpersonal sources (local authorities, health workers, neighbors/relatives) for information and adopted handwashing, mask-wearing, social distancing, and social restrictions to varying degrees. Overall, self-protective behaviors were largely positively and negatively associated with mass media and interpersonal sources, respectively, depending on the source-measure combination. Mistrust of the government seems to have shaped how Indigenous and mestizo peoples distinctively responded to interpersonal information sources and relied on mass media.

**Conclusions:**

Our findings call for improved media access to better manage pandemics in rural areas, especially among remote Indigenous communities.

**Supplementary Information:**

The online version contains supplementary material available at 10.1186/s12889-022-13772-y.

## Background

Understanding people’s reactions to the COVID-19 pandemic in low- and middle-income countries is critical in designing effective policies to address future pandemics and post-pandemic problems [1–6]. In remote rural regions where people have limited access to transportation, communication, and health services, available data are scant [7]. Self-protective measures are known to be critical in suppressing the contagion of the virus [8–12] and health risk communication plays a key role in diffusing such measures [13–19]. To date, little is known about the communication channels used by rural poor populations to learn novel measures and how effective these channels are for promoting self-protective behaviors.

Research has shown that trust in government institutions has been an important determinant of people’s compliance with public health policies during the Ebola epidemic in Africa [20, 21] and during the COVID-19 pandemic [22–25]. Institutional trust can differ significantly by social identity (e.g., ethnicity) and authorities (e.g., government vs. traditional leaders), underlying people’s distinct behavioral responses [26, 27]. As such, institutional mistrust among vulnerable rural populations may shape their behavioral responses to communication channels.

Our study examined COVID-19 health communication in remote rural communities in the Peruvian Amazon. Immediately after a national lockdown was lifted, we conducted telephone surveys with over 460 communities [28]. Peru ranks among the countries most severely affected by COVID-19, despite instituting one of the earliest and longest lockdowns in Latin America [29–32]. In many countries including Peru, indigeneity is a politically relevant social identity related to institutional trust. Our surveys covered both Indigenous and mestizo communities. Mestizos (folk peoples; locally known as *ribereños*) are descendants over many generations of Iberian and Indigenous peoples living in the region [33]. Concerns about the fate of Indigenous peoples, especially in Amazonia, are warranted and have been prominent in media reports and research on COVID-19, but tend to overlook the larger mestizo population [34–36].

Our study is novel in three important ways. This is the first report on the association of information sources about self-protective measures against COVID-19 with adoption of those measures in remote rural areas in developing countries. Most extant studies have been conducted in industrialized countries and China [13]. Second, we compare different media and interpersonal information sources over time, finding important contrasts in their potential effectiveness for the diffusion of self-protective measures [37–39]. Third, distinct from other telephone surveys on COVID-19 conducted at the household level, our large-scale telephone surveys were conducted at the community level which can better circumvent reporting bias (e.g., social desirability bias) and capture social behavior among people with distinct social identities over large geographical areas.

## Methods

### Setting

**Fig. 1:**
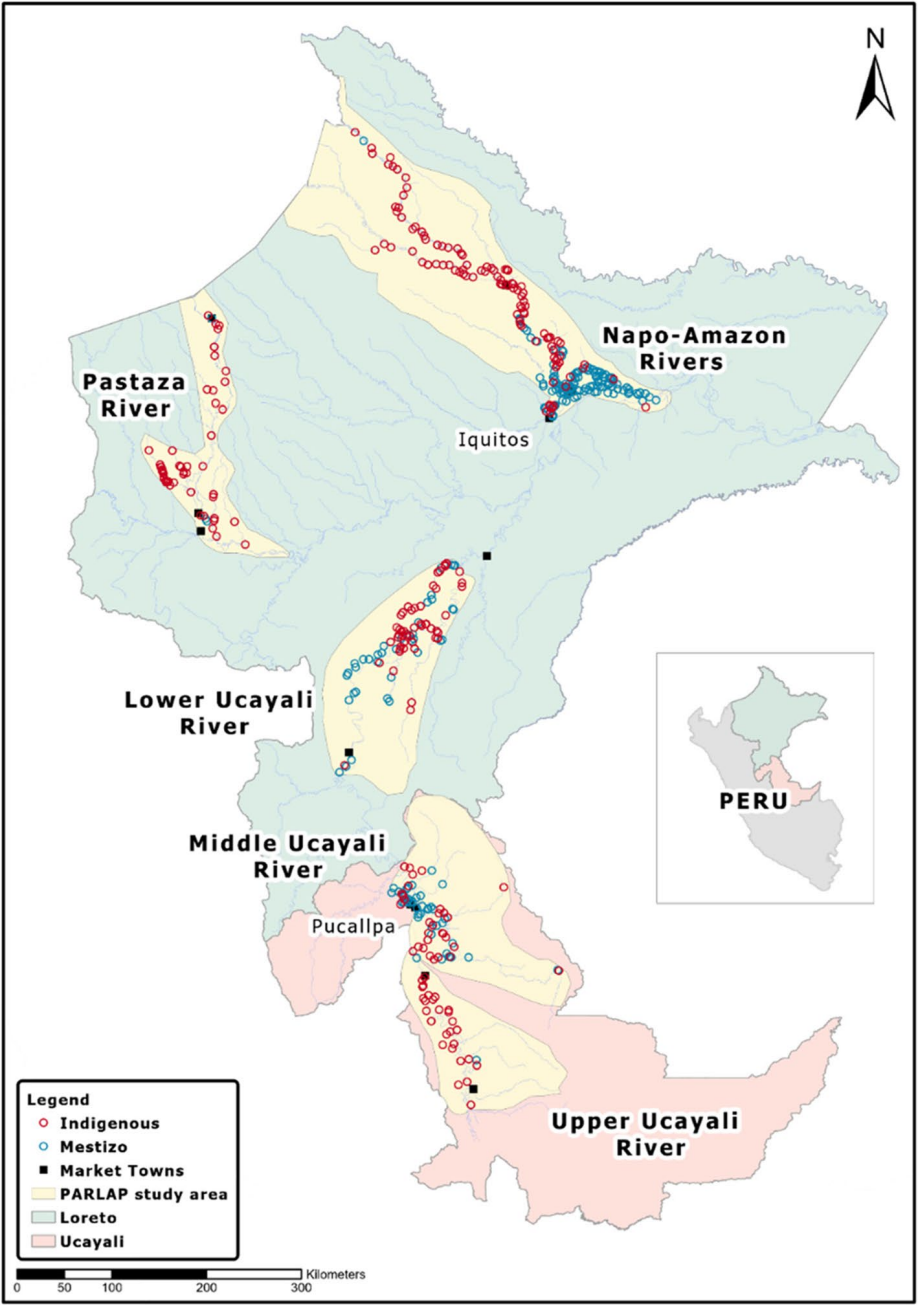
Surveyed communities

The Departments of Loreto and Ucayali, where our study area is located, cover about 85% of the area of the Peruvian Amazon (Fig. 1) consisting of humid tropical forest and extensive wetlands at < 200 m of elevation. Iquitos (population: 437,400) in Loreto and Pucallpa (population: 211,700) in Ucayali serve as major markets and administrative centers [40]. Iquitos can be reached only by river boat or by air; Pucallpa has also been connected with Lima, the capital city of Peru, by road since the 1940s. Small towns (5,000–30,000 inhabitants) and many smaller communities (100–300 inhabitants) line the main rivers and tributaries. Forest peoples (both Indigenous and mestizo) employ agriculture, fishing, hunting, timber and non-timber forest product gathering, and small livestock raising for subsistence and cash earnings, sending produce to market by boat [41–43].

In the Peruvian Amazon, COVID-19 spread in two waves in 2020 (April-June; August) and mortality was highest early during the first wave, especially in the cities of Iquitos and Pucallpa (Additional file 1: Fig. S1). A national lockdown which was declared mid-March 2020 lasted until early May, when restrictions were gradually relaxed [32]. By the end of June 2020, the lockdown was lifted, though various regional restrictions such as curfews were maintained.

### Surveys

Our COVID-19 surveys were part of the Peruvian Amazon Rural Livelihoods and Poverty (PARLAP) project (https://parlap.geog.mcgill.ca) [44]. We selected four major river basins – the Amazon, Napo, Pastaza, and Ucayali (nearly 120,000 km^2^, or about 2.4 times the area of Costa Rica; Fig. 1) – to capture the diversity of ecological conditions, economic activities, history, and indigeneity of its peoples. We sought to cover all communities in the study area. In each river basin, field teams were guided by maps from the Peruvian *Instituto Nacional de Estadística e Informática* (INEI) for the 2007 population census [40], the *Instituto del Bien Común* (IBC) for their census of Indigenous communities [45, 46], and Google Earth imagery, supplemented by local enquiries by the teams to identify unmapped settlements. The community survey conducted from December 2012 through March 2014 reached a total of 919 communities (436 Indigenous, 470 mestizo, and 13 colonist), which we estimate represents 92% of all communities in the study area (i.e., a near census). We use data from this community survey to construct some covariates.

Excluding district capitals and communities with a health center from 919 communities covered in the community survey, the remaining 893 communities were eligible for the COVID-19 survey [28]. Our baseline telephone survey conducted in July 2020 (between the two waves) covered 469 communities (53% of the target communities; 369 in Loreto, 100 in Ucayali). We subsequently conducted a follow-up telephone survey in August and early September 2020 (during the second wave) that reached 435 of the 469 communities in the baseline sample (7% attrition). The analysis sample is 466 communities at the baseline and 433 communities at the follow-up with no missing observations in key variables analyzed in the paper. Compared to mestizo communities, Indigenous communities are found in more remote areas in all river basins, especially in the Napo, Pastaza, and Upper Ucayali basins (Fig. 1). Indigenous peoples speak their own Indigenous language; many of them, especially young people, also speak Spanish [47, 48]. Mestizos generally speak Spanish only.

Our surveys sought information from community leaders, most of whom were males, following a structured questionnaire. With the suspension of public telephone service since November 2019 and an unreliable radiophone system, we relied mostly on cell phone contact. At the same time, our field teams visited ports and markets in Iquitos and Pucallpa to find people from the target communities. Some telephone interviews were arranged through an intermediary when people from the target communities visited a town where the intermediary lived. In these ways, distinct from standard telephone surveys, our surveys also contacted people in communities with no telephone access.

### Information and self-protective measures

**Fig. 2:**
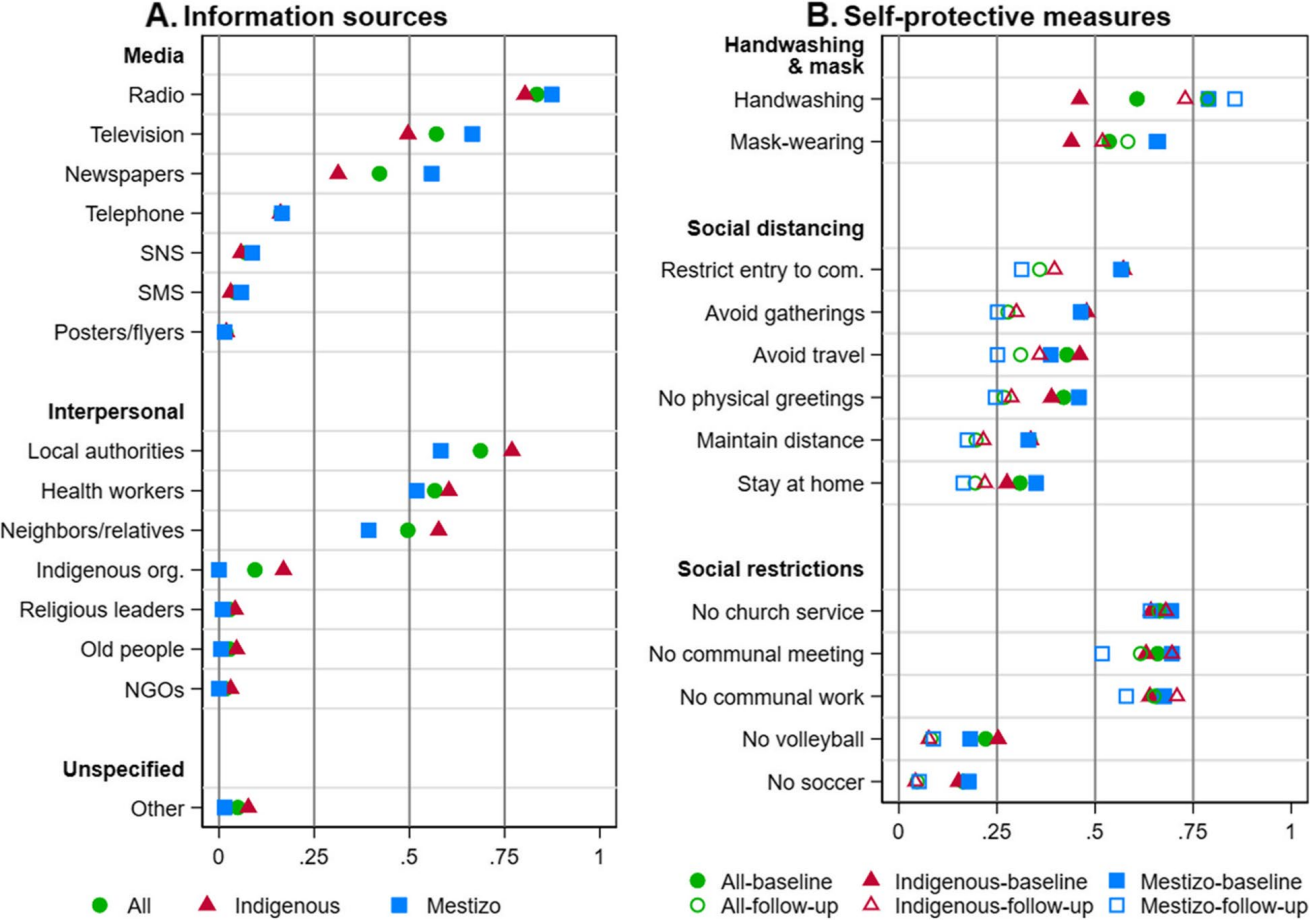
Information sources and self-protective measures against COVID-19. **A**. Proportions of communities using each source of information about self-protective measures in all, Indigenous, and mestizo communities at baseline. **B.** Proportions of communities using each self-protective measure in all, Indigenous, and mestizo communities at baseline and follow-up

The baseline survey asked whether people in the community had received any information about self-protective measures against COVID-19 at the time of interviews. Respondents in all communities answered “yes”. We then asked about information sources people used without specifying any sources (open-ended question). We did not restrict the number of answers. Respondents’ answers were recorded as posters/billboards/flyers, radio, television, short message service (SMS), phone, newspapers, social networking service (SNS) such as Facebook, Twitter, WhatsApp, etc. (media sources), health care workers, non-government organization (NGO) workers, Indigenous organization/federation, local authorities, neighbors/relatives, religious leaders, elders/people who know about traditional medicine (interpersonal sources), or other outreach. We construct indicator variables for each of these 15 information sources, which are not mutually exclusive (their means are reported in Fig. 2A).

The surveys asked (1) whether people in the community adopted handwashing, mask-wearing, and six social distancing measures – avoiding physical greetings, maintaining enough distance, staying at home, avoiding gatherings, avoiding travel, and restricting entry to the community – before the baseline survey (mid-March-July) and at the time of the follow-up survey; and, (2) whether the community restricted five social activities – playing soccer and volleyball, and gatherings for communal work, community meetings, and church services – during the previous 7 days at the time of the baseline and follow-up surveys. We construct indicator variables for each of these 13 individual measures, which are not mutually exclusive (their means are reported in Fig. 2B; an indicator variable for no church services takes 1 if there was no church in the community).

We capture the overall patterns of the adoption of multiple self-protective measures by constructing indices for each category of measures through principal component analysis based on the pooled baseline and follow-up data. Social distancing index is the first principal component of six social distancing measures. Social restriction index is the first principal component of five social restriction measures. Self-protective behavior index is the first principal component of handwashing, mask-wearing, six social distancing measures, and five social restriction measures. We standardize these indices as z-scores. In addition to the self-protective behavior index, which captures all original 13 measures, we consider handwashing, mask-wearing, and the social distancing and social restriction indices as outcomes. In this way, we can capture potential difference in adoption patterns among the latter four.

### Empirical design

We estimate the associations of information about self-protective measures against COVID-19 with adoption of those measures using Ordinary Least Squares (OLS) regression specifications of the form

1 $${Y}_{ict}=\mathrm{\alpha }+\upbeta \cdot {X}_{it}+\upgamma \cdot {Z}_{it}+{\phi }_{c}+{\varepsilon }_{ic}$$

where *Y*_*ict*_ is one of the five self-protective measures defined above of community *i* in basin *c* at period *t*; *X*_*it*_ is a vector of information sources; and *Z*_*it*_ is a vector of covariates, including interviewer fixed effects (8 interviewers); *ϕ*_*c*_ is a vector of basin fixed effects which capture basin heterogeneity (6 basins); and *ε*_*ic*_ is an error term. Inference is based on robust standard errors. We consider three specifications for information sources: the number of all sources, the number of media and interpersonal sources (two variables), and six indicator variables for primary sources defined below. Additional file 1: Table S1 shows the definition and descriptive statistics of covariates (the construction of some of them is provided in Additional file 1: Appendix A). The estimation results reported below are robust to various sets of covariates we considered. The number of observations for most dependent variables is slightly smaller than 466 at the baseline and 433 at the follow-up due to missing values. When we conduct heterogeneity analysis for primary information sources by indigeneity, we add an interaction term of an indicator variable for one of the sources and an indicator variable for Indigenous communities to Eq. (1).

## Results

### Information sources

Respondents reported 15 sources of information about self-protective measures against COVID-19 at the time of the baseline survey (Fig. 2A). Among seven media sources, radio was the most common (83%), followed by television (58%) and newspapers (42%). Telephone, SNS, SMS, and outdoor media (posters/flyers) were uncommon sources. For the seven interpersonal sources, local authorities (mostly in the community), health workers (including ones outside the community), and neighbors/relatives (including ones outside the community) were common (59–67%); Indigenous organizations, religious leaders, elders, and NGOs were uncommon sources of information. Other outreach (unspecified source) was also uncommon. Thus, three traditional mass media and three interpersonal sources were the primary information sources. Local authorities and health workers (formal interpersonal sources) who received information from the government/health authorities served as intermediaries between the government and people in the community. Neighbors/relatives (informal interpersonal sources) shared information with people in the community. We considered all possible combinations of the 15 sources, identifying 145 combinations in total. Among them, there were no dominant combinations. The most common combination – radio-newspapers-local authorities-health workers-neighbors/relatives (i.e., five primary sources) – was reported by only 6% of communities.

Nearly all communities (96%) had more than one information source and almost 80% of communities had more than two sources; having four sources – two media sources and two interpersonal sources – was the most common pattern (Additional file 1: Figs. S2A-C). The comparison of Indigenous and mestizo communities shows that the total number of information sources, especially interpersonal sources, was greater among the former (means: 4.2 vs. 3.9), though media sources (television and newspapers, in particular) were more common among the latter (Fig. 2A, Additional file 1: Figs. S2D-F). The spatial distribution of information sources is discussed in Additional file 1: Appendix B.

### Self-protective measures

According to the baseline survey, handwashing, mask-wearing (before the baseline survey; mid-March-July), and avoiding gatherings (for communal work, community meetings, and church services; during the previous 7 days) were employed by people in more than half of communities (54%-67%), followed by six social distancing measures (mid-March-July; 31%-57%); not-playing-sports (during the previous 7 days) was uncommon (Fig. 2B). Whereas handwashing and mask-wearing were more common among mestizo communities than Indigenous communities, social distancing and social restriction measures were similarly adopted in both types of communities (Fig. 2B). Correspondingly, overall self-protective behaviors (index) were stronger among mestizo communities than Indigenous communities, but both sets of communities adopted similar social distancing (index) and social restrictions (index) (Additional file 1: Figs. S3D, E, F).

Handwashing and mask-wearing, especially the former, became more common at the time of the follow-up, and this was so particularly among Indigenous communities (by 27%, almost 60% from baseline level), although both measures were still less common compared to mestizo communities (Fig. 2B). By the follow-up, social distancing (individual measures and index) became weaker, especially among mestizo communities (Fig. 2B, Additional file 1: Figs. S3B, E). In turn, social restrictions (individual measures and index) weakened among mestizo communities, but became more common among Indigenous communities because gatherings were more commonly avoided (Fig. 2B, Additional file 1: Fig. S3F); in the whole sample social restrictions changed little (Additional file 1: Fig. S3C). On balance, self-protective behaviors (index) became weaker at the follow-up among mestizo communities (Additional file 1: Fig. S3A, D). The spatial distribution and correlations of self-protective behaviors are discussed in Additional file 1: Appendices B and C.

### Associations of information sources with self-protective behaviors

**Fig. 3:**
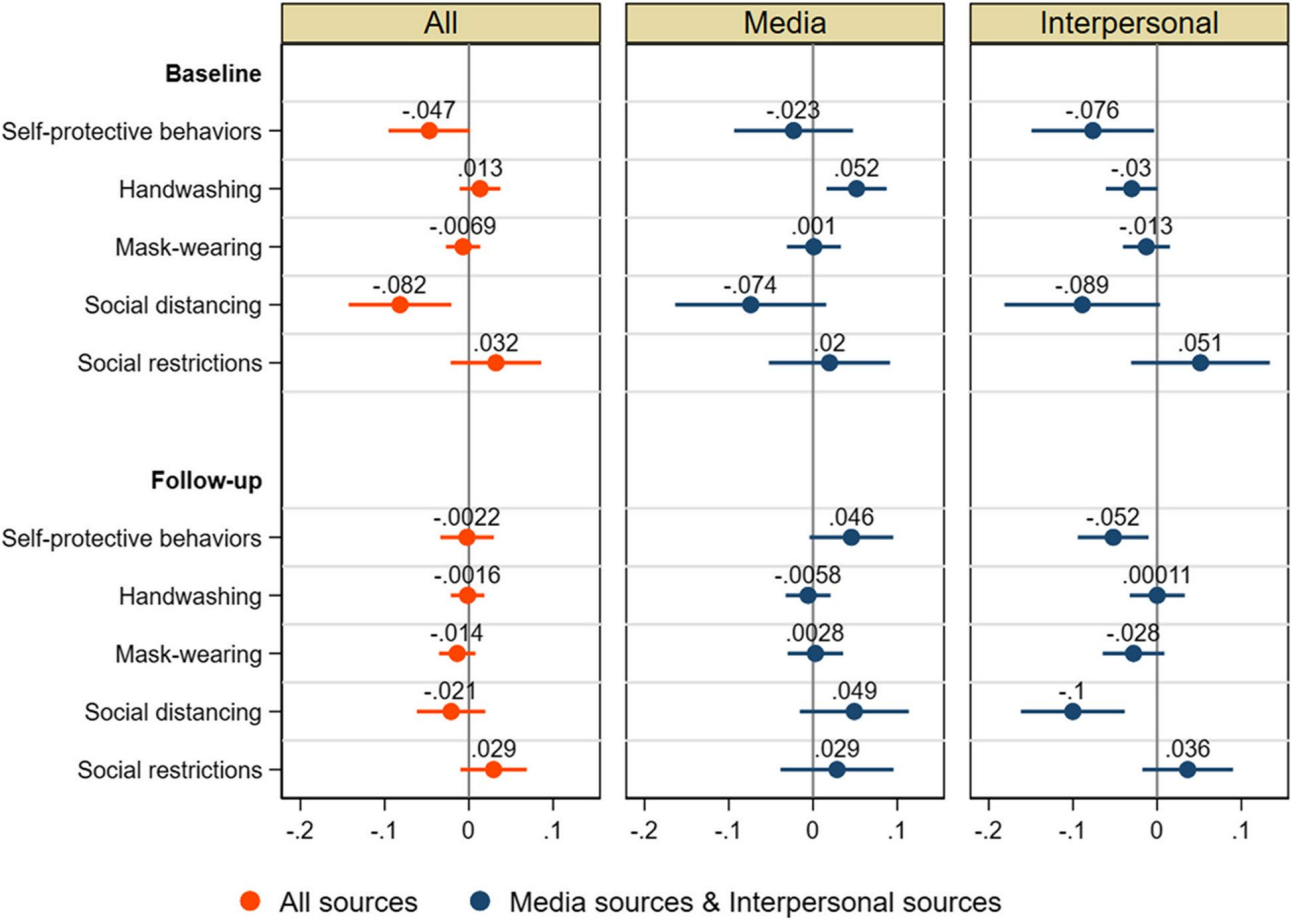
Associations of number of information sources with self-protective behaviors. OLS estimates of the coefficients of (1) number of all information sources and (2) number of media and interpersonal sources for self-protective behavior index (z-score), handwashing (0/1), mask-wearing (0/1), social distancing index (z-score), and social restriction index (z-score) at baseline and follow-up, with 95% confidence intervals based on robust standard errors

Figure 3 reports the OLS estimates of the coefficients of the number of information sources in Eq. (1) with a 95% confidence interval. The corresponding full regression results for the number of all sources are reported in Additional file 1: Table S2. The total number of information sources (out of 15) was associated with a reduction in self-protective behaviors (index), especially social distancing (by 0.08 SD per source), at the time of the baseline survey, but not the follow-up. Having three or more information sources (in comparison to one or two sources) was associated with a reduction in self-protective behaviors, especially social distancing, only at the baseline, and the associations were similar for three, four, five, and six or more sources (Additional file 1: Fig. S4). These results indicate that the relationship of the number of information sources with self-protective behaviors is nonlinear, because if their relationship was linear, the associations would be stronger in magnitude with a greater number of sources than three.

When we differentiate seven media and seven interpersonal sources, patterns are distinct. Whereas interpersonal sources were negatively associated with social distancing and self-protective behaviors at both baseline and follow-up, this relation for media sources with social distancing holds only at the baseline and the relation becomes positive at the follow-up (Fig. 3). The associations of the number of media/interpersonal sources with social distancing were similar regardless of the number of sources and these associations were similar to the association of any media/interpersonal sources with social distancing. Thus, what matters is whether people had at least one information source (media or interpersonal) (Additional file 1: Fig. S5). These results indicate that the associations with the number of information sources reported in Fig. 3 are shaped mainly by the type of media and interpersonal sources people obtained information from.

**Fig. 4:**
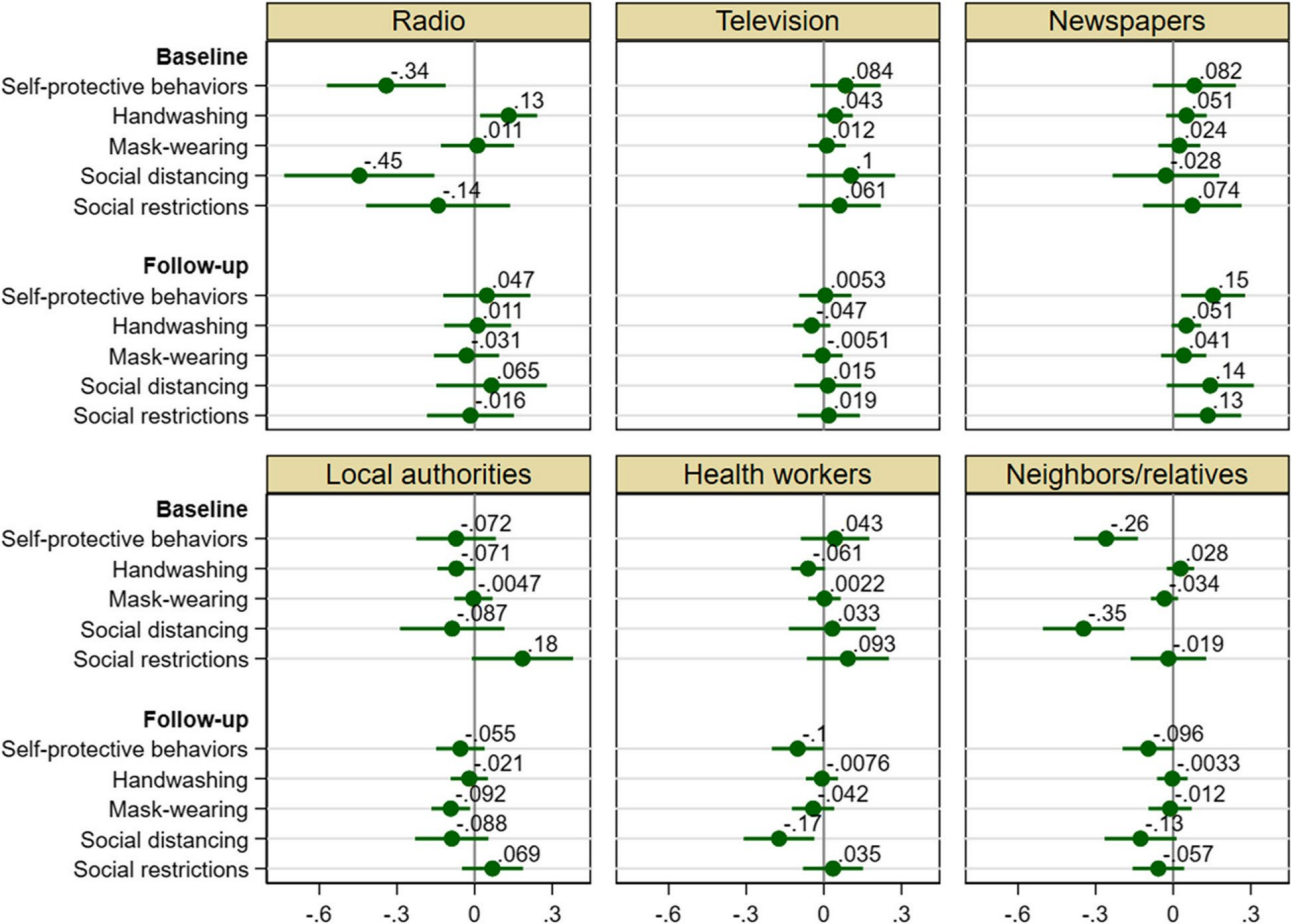
Associations of primary information sources with self-protective behaviors. OLS estimates of the coefficients of six indicator variables for primary information sources (radio, television, newspapers, local authorities, health workers, and neighbors/relatives) for self-protective behavior index (z-score), handwashing (0/1), mask-wearing (0/1), social distancing index (z-score), and social restriction index (z-score) at baseline and follow-up, with 95% confidence intervals based on robust standard errors

Figure 4 reports the OLS estimates of the coefficients of the six primary information sources. Among three primary media sources, (1) radio was negatively associated with self-protective behaviors, especially social distancing (by 0.45 SD), and positively associated with handwashing only at the baseline; (2) newspapers were positively associated with self-protective behaviors, especially social distancing, and social restrictions only at the follow-up; and, (3) no significant association was found for television at the baseline or follow-up (Fig. 4). Thus, the inverse associations found at the baseline and the follow-up for the number of media sources were mainly driven by radio and newspapers, respectively.

Among three primary interpersonal sources, (1) neighbors/relatives were negatively associated with self-protective behaviors, especially social distancing, over time (by 0.35 SD at the baseline), and the association significantly decreased (to 0.13 SD) over time; (2) local authorities were negatively associated with handwashing at the baseline and mask-wearing at the follow-up; and, (3) health workers were negatively associated with handwashing at the baseline, and social distancing and self-protective behaviors at the follow-up (Fig. 4). Thus, the negative associations of the number of interpersonal sources over time were driven by all three primary interpersonal sources. At the same time, local authorities were positively associated with social restrictions (by 0.18 SD) only at the baseline. The results for six original social distancing measures and five original social restriction measures are largely consistent (Additional file 1: Fig. S6).

### Heterogenous associations by indigeneity

**Fig. 5:**
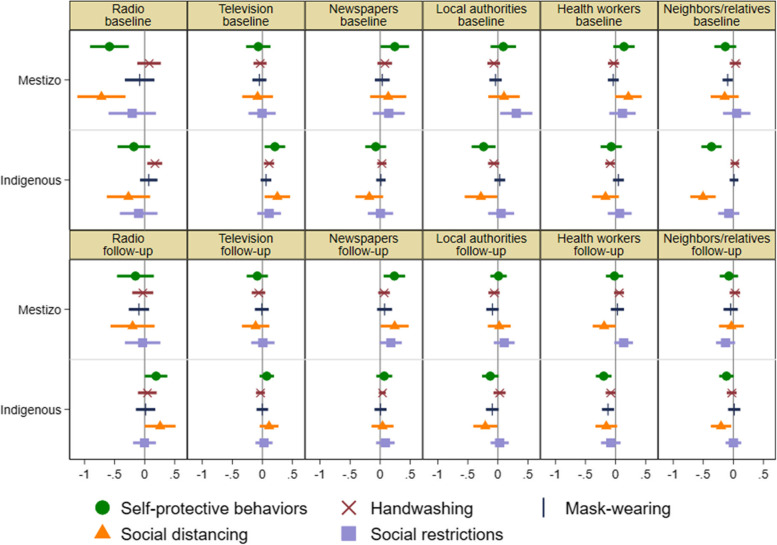
Associations of primary information sources with self-protective behaviors by indigeneity. OLS estimates of the coefficients of six indicator variables for primary information sources (radio, television, newspapers, local authorities, health workers, and neighbors/relatives) for self-protective behavior index (z-score), handwashing (0/1), mask-wearing (0/1), social distancing index (z-score), and social restriction index (z-score) at baseline and follow-up by indigeneity, with 95% confidence intervals based on robust standard errors

Figure 5 reports the OLS estimates of the coefficients of the six primary information sources by indigeneity. For mass media, we found that whereas radio’s negative association with social distancing at the baseline holds in both types of communities, the association was stronger in mestizo communities. Radio’s positive association with handwashing at the baseline holds only in Indigenous communities. In addition, radio was also positively associated with self-protective behaviors, especially social distancing, at the follow-up only in Indigenous communities. Newspapers were positively associated with self-protective behaviors over time only in mestizo communities (it was so with handwashing, social distancing, and social restrictions at the follow-up). In contrast, television was positively associated with self-protective behaviors, especially handwashing and social distancing, at the baseline only in Indigenous communities.

For interpersonal sources, we found that among Indigenous communities, none of the associations for local authorities and health workers were positive at both baseline and follow-up; indeed and surprisingly, they were mostly negatively associated with self-protective behaviors. Among mestizo communities, in contrast, both positive and negative associations were found at the follow-up though none of them were negative at the baseline: (1) positive and negative associations of health workers with social distancing at the baseline and follow-up, respectively (i.e., reversal); (2) positive associations of local authorities with social restrictions at the baseline and health workers with social restrictions and handwashing at the follow-up; and (3) negative associations of local authorities with handwashing and mask-wearing at the follow-up. Among both types of communities, none of neighbors/relatives’ associations were positive over time and negative associations were found for self-protective behaviors: (1) social distancing over time in Indigenous communities; and (2) mask-wearing at the baseline and social restrictions at the follow-up in mestizo communities. The association of self-protective behaviors at the baseline was stronger in Indigenous communities (the difference is statistically significant according to t-test, *p* = 0.068), although the association decreased significantly over time.

## Discussion

### Information sources

In the Peruvian Amazon, forest peoples relied on multiple media and interpersonal sources for information about self-protective measures against COVID-19 – radio, television, newspapers, local authorities, heath workers, and neighbors/relatives, in particular. Radio was the critical media source in remote rural communities. Although 45% and 14% of communities had cell phone and internet access, respectively, telephone and social media were uncommon information sources, reflecting limited access to and usage of communication technology among forest peoples.

Less common use of television and newspapers as information sources among Indigenous communities than mestizo communities likely indicates their limited access to a TV signal and print media via river transportation in remote locations. Indigenous peoples seem to have substituted interpersonal sources for external information sources; in particular, television was negatively correlated with local authorities and neighbors/relatives as information sources (Additional file 1: Fig. S7).

### Self-protective measures

People in rural communities adopted a range of self-protective measures, including individual practices with which they were not familiar prior (e.g., mask-wearing) and restrictions on community social activities that were common before the pandemic (e.g., communal work). Rural peoples had difficulties in maintaining social distancing and not playing sports. This likely reflects social and cultural norms, their housing environments, and lifestyle, though we cannot identify specific reasons with our data. Among Indigenous peoples, handwashing increased over time. Social distancing and social restrictions, however, decreased by the time of the follow-up, especially among mestizos.

### Associations of information sources with self-protective behaviors

Radio’s strong initial negative association with social distancing could be explained by factors such as misinformation, confusion, or inattention at initial stages of the pandemic; information broadcasted from some radio stations might have discouraged social distancing. Although such problems with audio media could be exacerbated by linguistic constraints for Indigenous peoples [49], the negative association was stronger among mestizos, suggesting that language was not a major barrier.

Among Indigenous peoples, radio’s association with social distancing subsequently became positive and television’s initial association was also positive. For both radio and television, the initial association with handwashing was positive. Thus, although both types of media were effective for promoting the simple habitual practice of handwashing, the salience of visual information mattered for unfamiliar social distancing at the initial stage of the pandemic, and once people’s understanding improved, radio also became effective, which buttresses the idea of limited linguistic constraints. The positive correlation between radio and television was consistently stronger among Indigenous peoples than mestizos (Additional file 1: Fig. S7).

In contrast, newspaper’s positive association with self-protective behaviors over time (social distancing and social restrictions at the follow-up) among mestizos may suggest the potential advantage of print media for information processing (e.g., comprehension through reading, referencing) among Spanish-speaking populations. Then, linguistic constraints might be stronger for print media than audio/visual media among Indigenous peoples for whom Spanish would be a second language. Newspapers were more common in the Amazon and Napo basins near Iquitos (Additional file 1: Fig. S8C), indicating underlying geographical heterogeneity.

The initial negative and positive associations of local authorities and health workers with some self-protective measures among Indigenous peoples and mestizos, respectively, are consistent with a general sense of mistrust of the government that prevails among Indigenous peoples [36, 50–53]. At the initial stage of the pandemic, under enormous uncertainty, Indigenous peoples may have felt that adopting novel social distancing measures following these formal interpersonal sources could hurt rather than help them, as experienced elsewhere with the Ebola virus [21]. Increased negative associations of the formal interpersonal sources among mestizos over time may suggest that their mistrust increased during the pandemic; in particular, the considerable association of health workers with social distancing reversed.

The contrast between Indigenous peoples and mestizos regarding mass media may also be related to low levels of trust in the government. Although Indigenous peoples with strong mistrust relied on radio and television over time, mestizos did not do so initially possibly because they relied on formal interpersonal sources instead, and as their mistrust in government increased over time, they may have not trusted radio or television either, while they continued to rely on newspapers.

The negative associations of informal interpersonal sources (neighbors/relatives) may have arisen because of misunderstandings, confusion, peer effect, and so forth. The stronger initial negative associations among Indigenous peoples than mestizos could be related to media and formal interpersonal sources: neighbors/relatives were positively correlated with local authorities in Indigenous communities and newspapers in mestizo communities (which were negatively and positively associated with self-protective behaviors, respectively; Additional file 1: Fig. S7). Decreased negative associations of neighbors/relatives especially in Indigenous communities over time likely reflects people’s improved understanding of self-protective measures.

### Limitations

Our study has three important limitations. First, we lack data on the content of information about self-protective measures against COVID-19 people received from each information source. Since our study did not focus on any communication campaigns, it was infeasible to ask about specific content in a systematic way in our brief telephone surveys. The actual information content from the same source, especially mass media, could vary across information providers. For example, information from radio could be considerably different across radio stations, as conjectured speculated above. The specific content of information people received from the same source could also vary among people within the same community, which our community surveys could not capture. The lack of information content constrained our assessment of the effectiveness of information sources.

A second limitation is the lack of data on people’s trust of the government. We did not include questions about trust at the community level, because it was infeasible to do so in a way that would limit potential reporting bias in our brief telephone surveys. Institutional trust could significantly vary among people within the same community, which our community surveys could not capture. Without trust data, we are limited in the ability to interpret our results, especially regarding the negative associations of self-protective behaviors with information sources. Relatedly, our data do not allow us to examine how cultural and social norms may underlie the different patterns observed between Indigenous and mestizo peoples. Our community-level survey data also do not allow us to explore household-/individual-level factors such as gender.

Finally, our study presents issues related to external validity, which has been a common problem of telephone surveys during the pandemic. Even though our surveys covered many communities without telephone access, our analysis samples are not entirely representative of the PARLAP study area as discussed in Additional file 1: Appendix D and, as such, may not be generalizable to the entire PARLAP study area.

## Conclusion

Our findings on COVID-19 information and self-protective behaviors in rural communities in the Peruvian Amazon have the following implications for research and policy. First, in the Internet era, rural peoples with limited communication access still rely on traditional media and interpersonal sources to receive information about self-protective measures. Under such circumstances, conventional information sources need to receive attention for effective policymaking to manage pandemics in rural areas.

Second, traditional media can be a potentially effective channel for promoting some but not all self-protective measures. Radio can be effective for simple habitual practices (e.g., handwashing). This is important because radio can be a primary media source in remote locations. However, audio media may backfire for ‘tough’ social distancing measures at an initial stage of pandemics when people have limited understanding of pandemics. To this end, visual media (television) and print media (newspapers) could be more effective for different reasons (visual salience and information processing).

Third, whether formal interpersonal sources (e.g., local authorities, health workers) can serve as effective intermediaries between the government and people in rural communities depends on people’s level of trust in government. For populations with weak trust in the government such as Indigenous peoples in Peru [36, 50–53], formal interpersonal sources such as health workers can be problematic. When government responses to pandemics are ineffective, this problem may worsen over time as institutional trust deteriorates. Information sharing through informal interpersonal sources (e.g., neighbors/relatives) can also be problematic until people develop a basic understanding of pandemics. With limited access to mass media, however, rural people tend to rely on such interpersonal sources. Improving media access in rural areas is thus critical for better communication under pandemics. In Amazonia, this is especially so among Indigenous communities where wariness of the government is common in remote areas as is vaccine hesitancy.

All of the above implications apply also to communication channels for promoting COVID-19 vaccination [54]. With weak institutional trust among people, effective communication through mass media is of critical importance. Community-level analysis of information and self-protective behaviors including vaccination can serve as a first-order analysis of health risk communication especially when conducted over large geographical areas. This approach is practical for rural research during pandemics when researchers must rely on telephone surveys to collect data.

## Supplementary Information

**Additional file 1.**

## Data Availability

The datasets used and/or analysed during the current study are available from the corresponding author on reasonable request.
