# Supplementary material for: COVID-19 information and self-protective behaviors among rural communities in tropical forests
Source: BMC Public Health. 2022 Jul 20;22:1394. doi: 10.1186/s12889-022-13772-y (PMC9299405; doi:10.1186/s12889-022-13772-y)
Supplement: Supplementary file 1 — Additional file 1. [file 12889_2022_13772_MOESM1_ESM.pdf]

Additional file 1
Supplementary Information

COVID-19 information and self-protective behaviors among rural communities in tropical forests

Yoshito Takasaki*, Christian Abizaid, Oliver T. Coomes

*Corresponding author. Email: takasaki@e.u-tokyo.ac.jp

June 2, 2022

Contents

Appendix A. Construction of covariates 2
Appendix B. Spatial distribution of information sources and self-protective behaviors 2
Appendix C. Correlations of individual self-protective measures 2
Appendix D. Sample representativeness 3
Fig. S1. Regional trends of COVID-19 case and mortality 4
Fig. S2. Histogram plots of number of sources of information about self-protective measures against COVID-19 5
Fig. S3. Distributions of self-protective behaviors 6
Fig. S4. Nonlinear relations of number of information sources with self-protective behaviors 7
Fig. S5. Nonlinear relations of number of media and interpersonal sources with self-protective behaviors 8
Fig. S6. Associations of primary information sources with individual self-protective measures 9
Fig. S7. Heat plots of correlations of primary information sources 10
Fig. S8. Primary information sources and self-protective behaviors across communities 11
Fig. S9. Heat plots of correlations of individual self-protective measures 12
Table S1. Definition and descriptive statistics of covariates 13
Table S2. OLS estimates for number of all information sources 14
Table S3. Sample representativeness 16

Appendix A. Construction of covariates

- Distance from city – River network distance [1] from Iquitos or Pucallpa (closer one) to each community.
- Active public river transportation – At least one public river boat (large river boat (*lancha*), small river boat (*colectivo*), or speed boat (*rápido*)) [2, 3] during the previous 7 days at the time of the baseline/follow-up surveys.
- Inactive public river transportation – No public river boat during the previous 7 days at the time of the baseline/follow-up surveys among communities with access to public river transportation before the COVID-19 pandemic.
- Distance from nearest market town – River network distance [1] from nearest market town out of 13 (including ones outside the study area) to each community.
- Distance from nearest community – River network distance [1] from the nearest community out of all other communities covered in the community survey to each community.
- Floodplain soils – A proxy for young alluvial soils in floodplain. The proportion of land area in a 5 km buffer centered on the community that is underlain by Holocene parent material. Based on *La Carta Geológica Nacional Mapa Geológico del Peru* (1:100,000) published by INGEMMET (Instituto Geológico Minero y Metalúrgico, Lima); available on-line at: https://portal.ingemmet.gob.pe/web/guest/carta-geologica-nacional-escala-1-100-000. Simplified reclassification of INGEMMET's 'Soil_NAME' variable, as soils being formed during the Holocene period (Qh), Pleistocene (Qp), or earlier (Tertiary).
- Forest – The proportion of land area in a 5 km buffer centered on the community in 2015 which was classified from Landsat imagery to be forest with CLASlite™ v3.2. [4]

Appendix B. Spatial distribution of information sources and self-protective behaviors

Most communities that did not use radio as information source are located in the Pastaza or Lower Ucayali basins (Fig. S8A). Television and newspapers were more common among mestizo communities than Indigenous communities; in particular, television was uncommon and newspapers were nonexistent in the Pastaza basin (Fig. S8B, C). Newspapers were uncommon also among mestizo communities in the Middle and Upper Ucayali basins. These patterns are likely to reflect the limited access to a TV signal and print media via river transportation in remote locations, especially among Indigenous communities. Interpersonal sources (local authorities, health workers, neighbors/relatives) were more common among Indigenous communities than mestizo communities; in particular, health workers were uncommon in the Pastaza basin (Fig. S8E-G).

Overall, self-protective behaviors (index) were strong in the Napo basin at both baseline and follow-up, those among mestizo communities in the Amazon basin were relatively weak at the follow-up compared to the baseline, and the distributions in other basins were similar over time (Fig. S8D, H). These patterns indicate that the weakening of self-protective behaviors among mestizo communities over time was significant along the Amazon River.

Appendix C. Correlations of individual self-protective measures

The correlations of 11 individual self-protective measures (excluding uncommon not-playing-sports) show that (1) almost all measures were positively correlated with each other; (2)

the correlations were stronger in Indigenous communities than mestizo communities at the baseline; (3) the correlations became stronger at the follow-up in mestizo communities and in the whole sample; (4) closely related measures (e.g., handwashing and mask-wearing; no physical greetings and maintaining distance; three no-gathering measures) were strongly correlated with each other; (5) handwashing and mask-wearing were strongly correlated with social distancing and non-gathering measures in Indigenous communities especially at the baseline; and, (6) social distancing and non-gathering measures were correlated with each other in both Indigenous and mestizo communities, especially at the follow-up (Fig. S9).

Appendix D. Sample representativeness

We examine whether our non-randomly sampled communities in the COVID-19 surveys are representative of the PARLAP study area (Table S3). On one hand, the analysis samples were not correlated with telephone access, distance from cities, access to public river transportation, or the availability of health facility. On the other hand, Indigenous communities and communities in the Napo basin (the Middle and Upper Ucayali basins), far from market towns, and with past NGO activities, are more (less) likely to have been sampled in the baseline survey and been found in the follow-up survey.

References

1. Webster K, Arroyo-Mora JP, Coomes OT, Takasaki Y, Abizaid C. A cost path and network analysis methodology to calculate distances along a complex river network in the Peruvian Amazon. Appl Geogr. 2016;73:13-25.
2. Salonen M, Toivonen T, Cohalan J-M, Coomes OT. Critical distances: Comparing measures of spatial accessibility in the riverine landscapes of Peruvian Amazonia. Appl Geogr. 2012;32(2):501-13.
3. Abizaid C, Coomes OT, Takasaki Y. Lifeways and currents of change in the Peruvian Amazon: A 1000 km boat journey down the Ucayali River. Focus Geogr. 2022;65.
4. Asner GP, Knapp DE, Balaji A, Páez-Acosta G. Automated mapping of tropical deforestation and forest degradation: CLASlite. J Appl Remote Sens. 2009;3(1):033543.

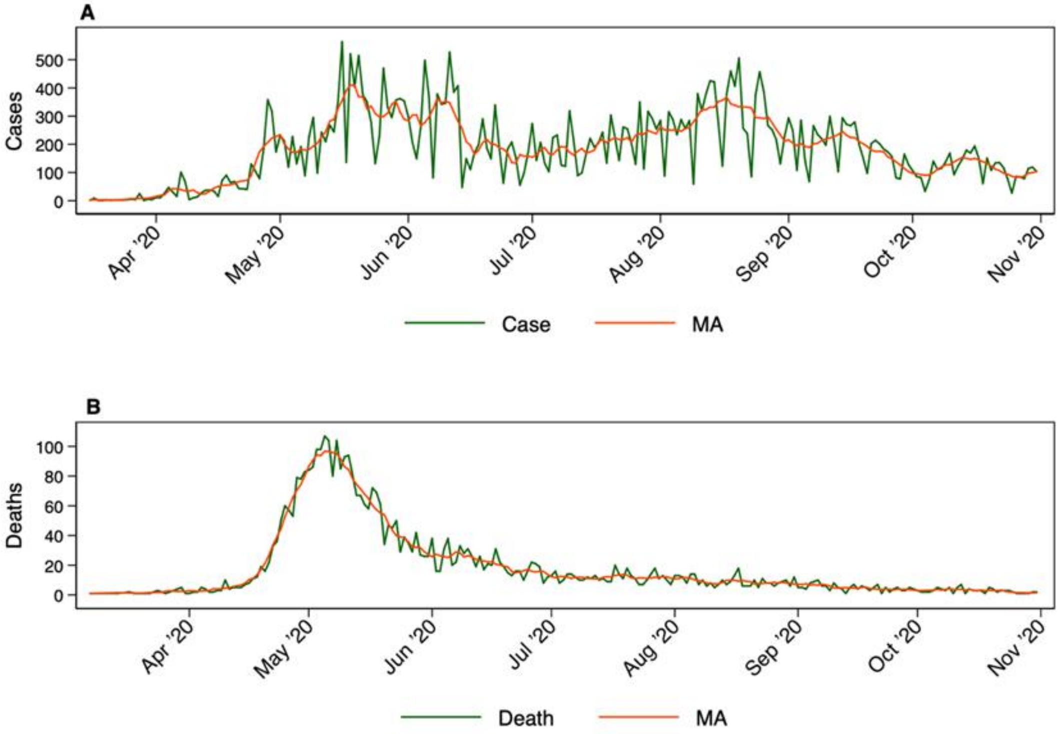

Fig. S1. Regional trends of COVID-19 case and mortality. A. Daily confirmed cases of COVID-19 in Loreto and Ucayali with symmetric 7-day moving average (MA). **B.** Daily mortality due to COVID-19 in Loreto and Ucayali with symmetric 7-day moving average (MA). Source: Peruvian Ministry of Health (Ministerio de Salud, MINSA), https://www.datosabiertos.gob.pe/group/datos-abiertos-de-covid-19, accessed June 16, 2021.

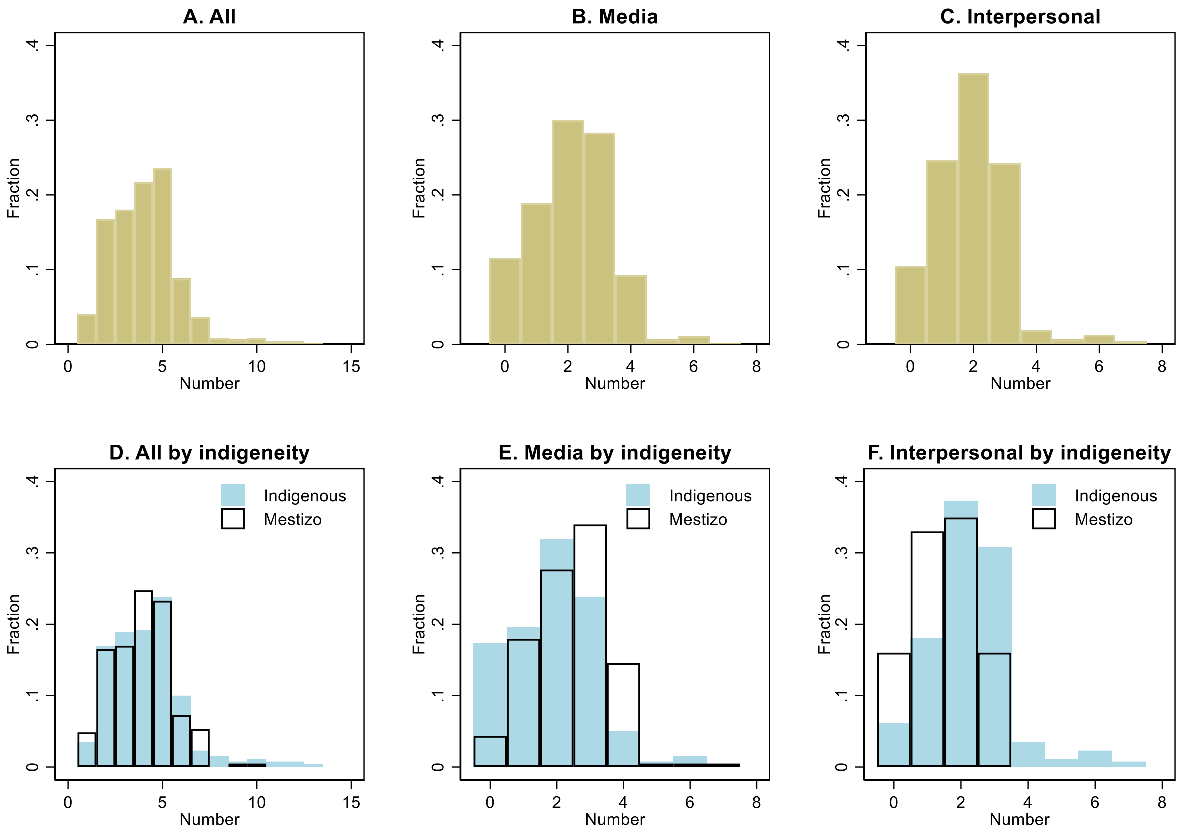

Fig. S2. Histogram plots of number of sources of information about self-protective measures against COVID-19. Fractions are shown. **A.** All information sources. **B.** Media sources. **C.** Interpersonal sources. **D.** All information sources by indigeneity. **E.** Media sources by indigeneity. **F.** Interpersonal sources by indigeneity.

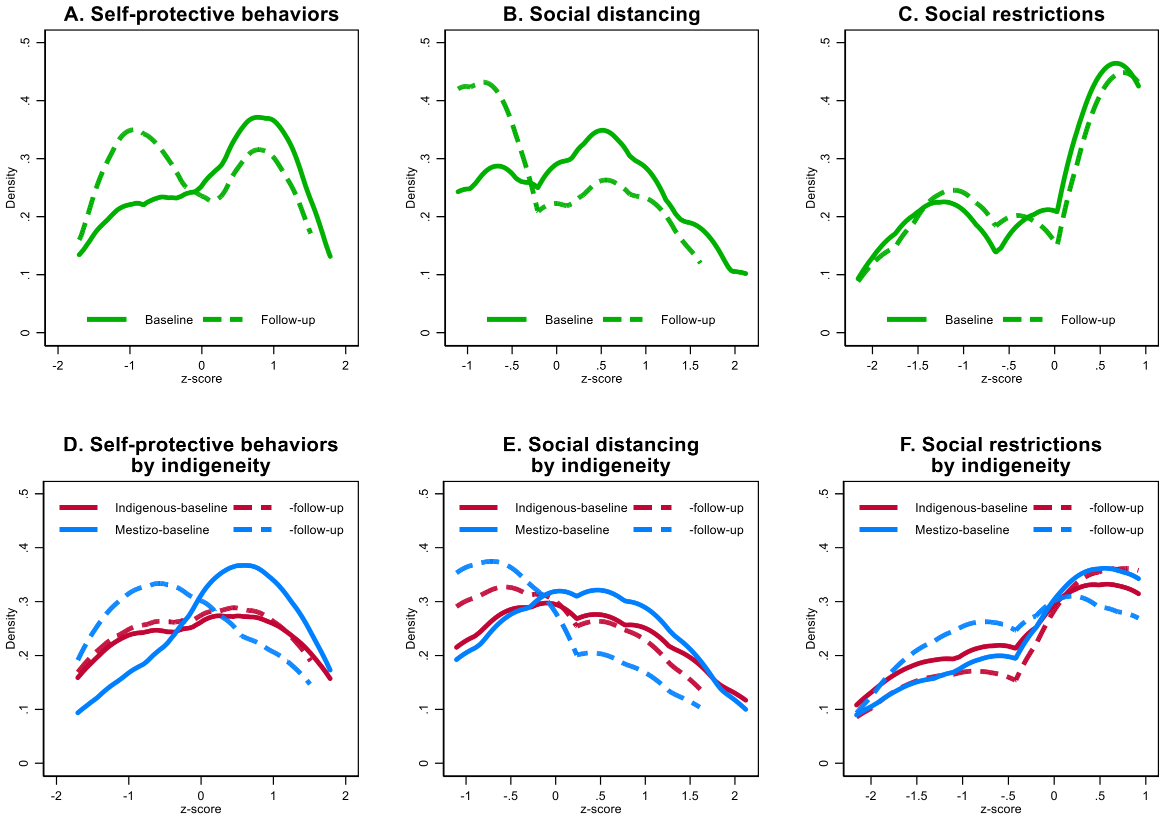

Fig. S3. Distributions of self-protective behaviors. The estimated densities of self-protective behavior indices (z-score) in the whole sample (**A**) and by indigeneity (**D**); social distancing indices (z-score) in the whole sample (**B**) and by indigeneity (**E**); social restriction indices (z-score) in the whole sample (**C**) and by indigeneity (**F**), at baseline and follow-up.

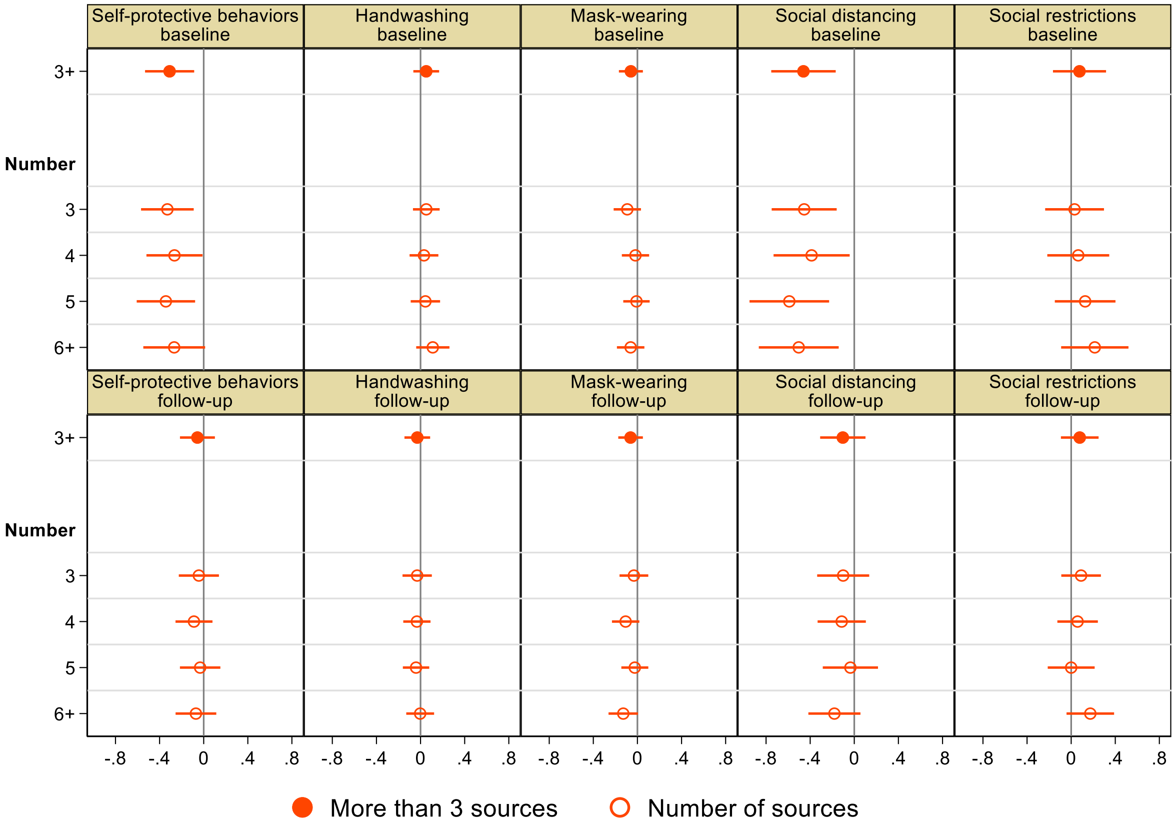

Fig. S4. Nonlinear relations of number of information sources with self-protective behaviors. The OLS estimates of the coefficients of (1) an indicator variable for three or more information sources and (2) four indicator variables for three, four, five, and six or more information sources for self-protective behavior index (z-score), handwashing (0/1), mask-wearing (0/1), social distancing index (z-score), and social restriction index (z-score) at baseline and follow-up, with 95% confidence intervals based on robust standard errors.

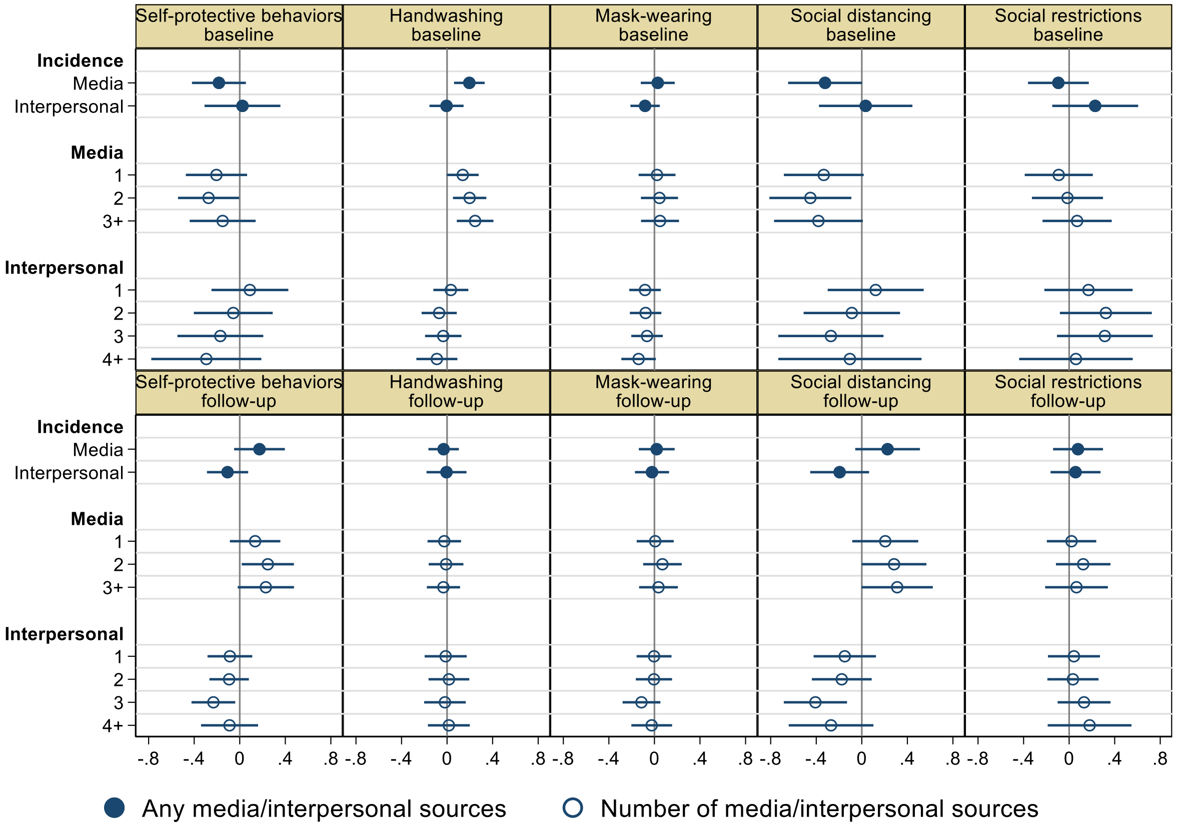

Fig. S5. Nonlinear relations of number of media and interpersonal sources with self-protective behaviors. The OLS estimates of the coefficients of (1) two indicator variables for any media and any interpersonal sources and (2) three indicator variables for one, two, and three or more media sources and four indicator variables for one, two, three, and four or more interpersonal sources for self-protective behavior index (z-score), handwashing (0/1), mask-wearing (0/1), social distancing index (z-score), and social restriction index (z-score) at baseline and follow-up, with 95% confidence intervals based on robust standard errors.

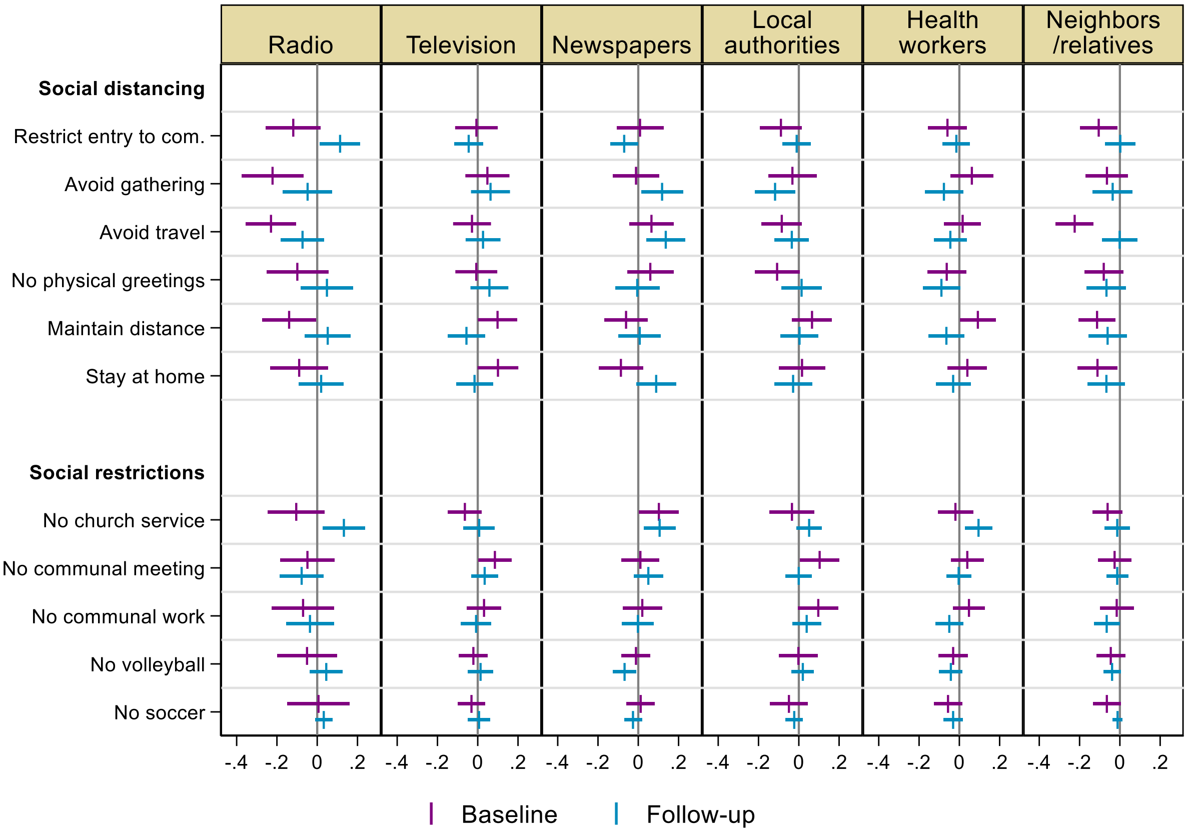

Fig. S6. Associations of primary information sources with individual self-protective measures. The OLS estimates of the coefficients of six indicator variables for primary information sources (radio, television, newspapers, local authorities, health workers, and neighbors/relatives) for six social distancing measures (0/1) and five social restriction measures (0/1) at baseline and follow-up, with 95% confidence intervals based on robust standard errors.

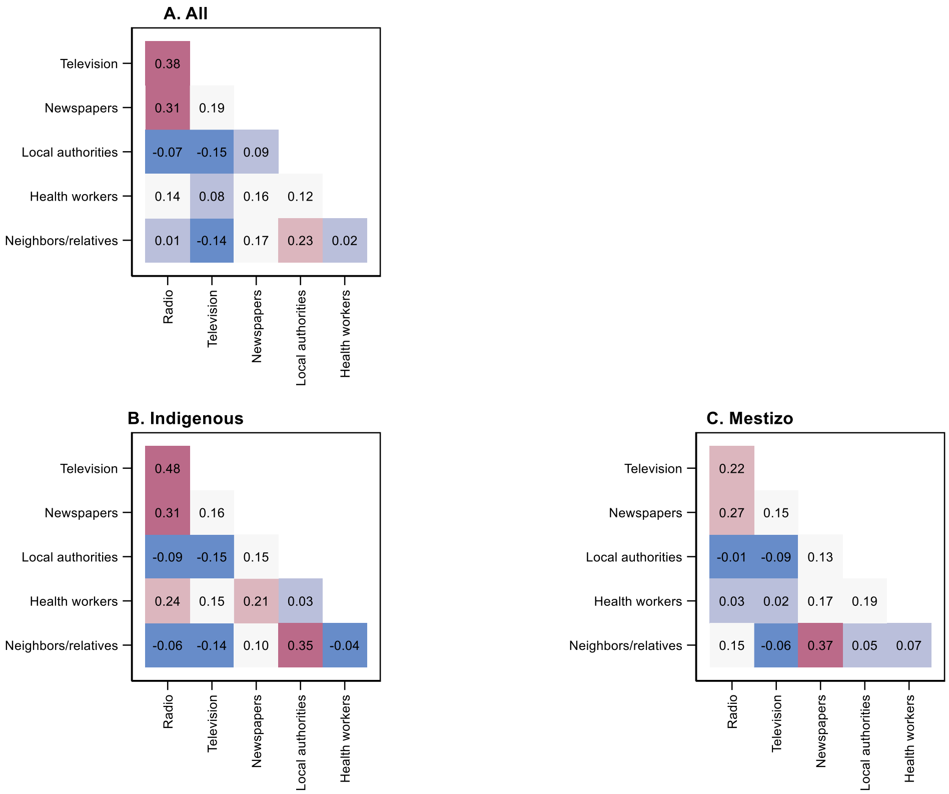

Fig. S7. Heat plots of correlations of primary information sources. Correlation coefficients (*r*) of six indicator variables for primary information sources (radio, television, newspapers, local authorities, health workers, and neighbors/relatives), using a color gradient, in all (**A**), Indigenous (**B**), and mestizo (**C**) communities at baseline. $p<0.05$ for $|r|>0.07$ (A) and $|r|>0.10$ (B, C).

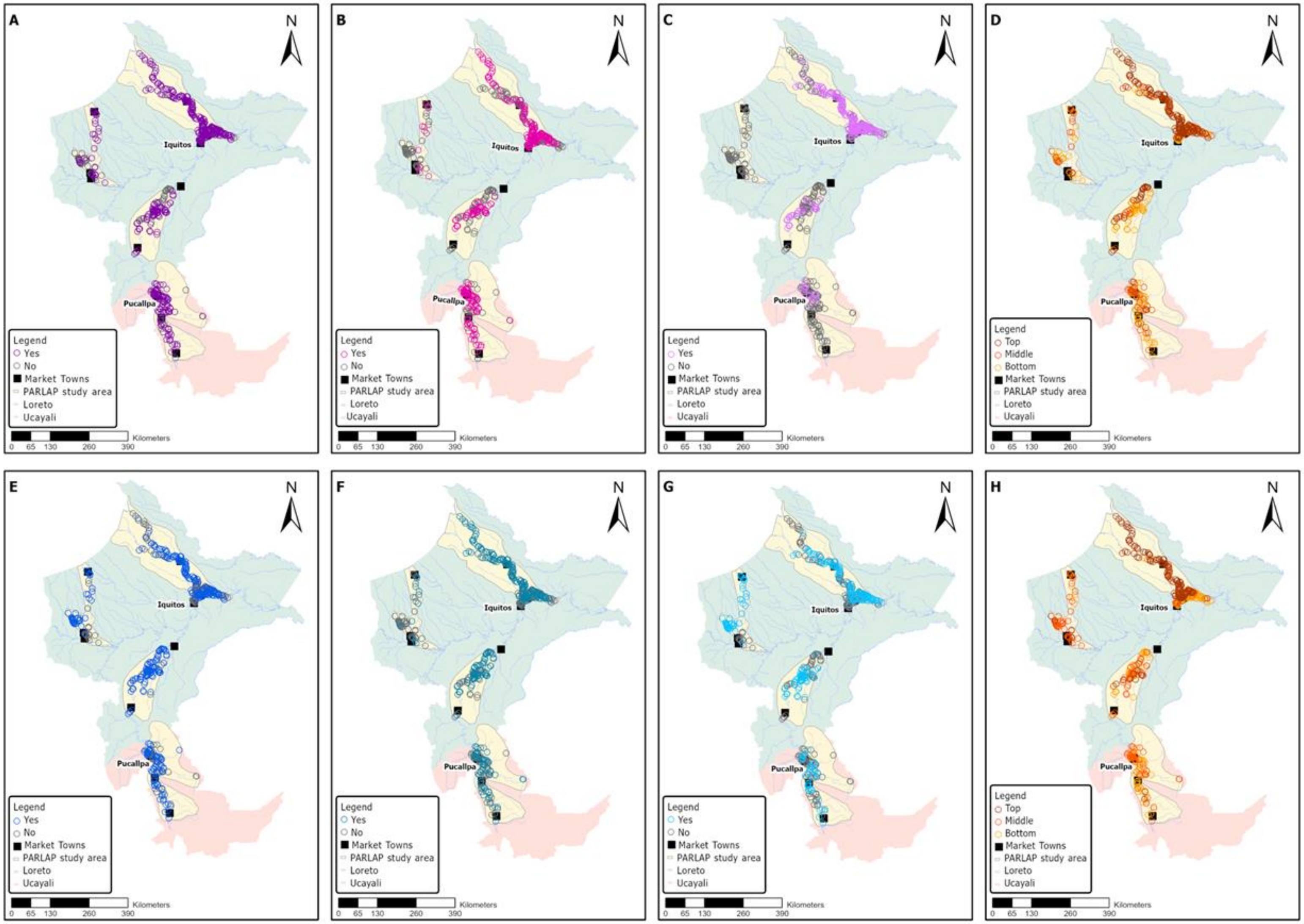

Fig. S8. Primary information sources and self-protective behaviors across communities. An indicator variable for radio (**A**), television (**B**), newspapers (**C**), local authorities (**E**), health workers (**F**), and neighbors/relatives (**G**) at baseline. Terciles for self-protective behavior index (z-score) at baseline (**D**) and follow-up (**H**).

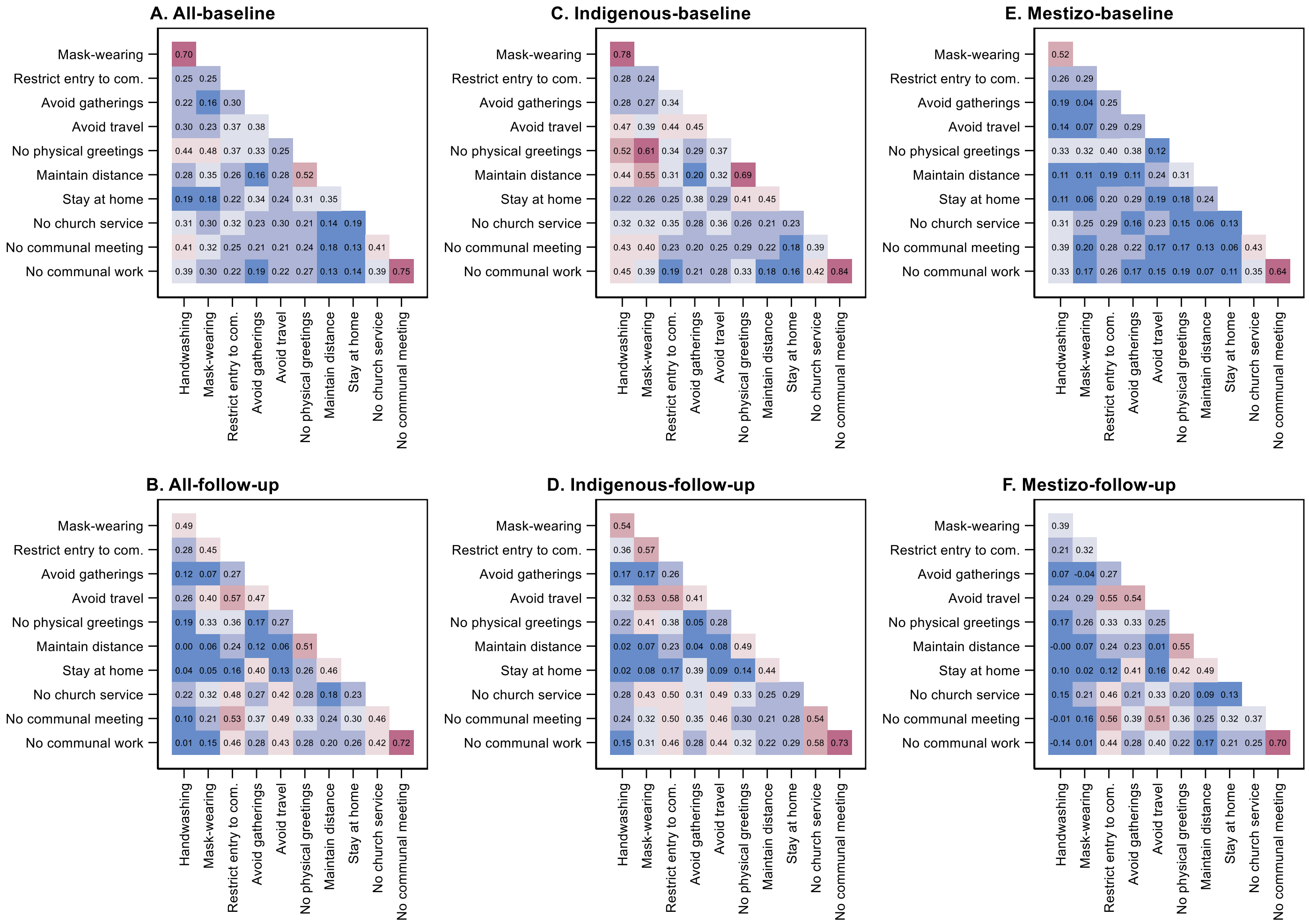

Fig. S9. Heat plots of correlations of individual self-protective measures. Correlation coefficients (*r*) of handwashing, mask-wearing, six social distancing measures, and three social restriction measures (0/1), using a color gradient, in all (**A**), Indigenous (**C**), and mestizo (**E**) communities at baseline, and in all (**B**), Indigenous (**D**), and mestizo (**F**) communities at follow-up. $p<0.05$ for $|r|>0.10$ (A, B) and $|r|>0.14$ (C-F).

Table S1. Definition and descriptive statistics of covariates

Variables	Definition	Mean	SD	Mean	SD
Indigenous (0/1)	Indigenous community (self-report)[a,b]	0.56	0.50	0.55	0.50
Population (log)	Number of inhabitants at baseline survey	5.16	0.96	5.16	0.96
Distance from city (log km)	River network distance from Iquitos or Pucallpa (closer one)	5.08	1.11	5.05	1.12
Active public river transportation (0/1)	At least one public river boat during the previous 7 days at the time of interviews	0.65	0.48	0.67	0.47
Inactive public river transportation (0/1)	Interruption of public river boat during the previous 7 days at the time of interviews	0.15	0.36	0.13	0.33
Communication acces (0/1)	Internet access, cell phone access, or availability of radiophone	0.75	0.44	0.74	0.44
Health facility (0/1)	Community has a health post	0.20	0.40	0.20	0.40
Past epidemic (0/1)	Past experience of empidemic (e.g., cholera) before the COVID-19 pandemic[b]	0.84	0.37	0.83	0.37
Past NGO activities (0/1)	Engagement of NGOs since 1990 before the COVID-19 pandemic[b]	0.52	0.50	0.52	0.50
Past health NGO activities (0/1)	Engagement of health NGOs since 1990 before the COVID-19 pandemic[b]	0.13	0.33	0.12	0.33
Secondary school (0/1)	Community has a secondary school	0.30	0.46	0.29	0.46
Church (0/1)	Community has a church	0.85	0.36	0.85	0.36
Population nearby (log)	Number of inhabitants in 5km buffer[b,c]	5.45	1.25	5.46	1.27
Distance from nearest market town (log km)	River network distance from nearest market town	3.95	1.09	3.93	1.09
Distance from nearest community (km)	River network distance from nearest community	5.63	6.72	5.59	6.87
Community market (0/1)	All five commodities (rice, sugar, cooking oil, soap, batteries) can be bought in community[b]	0.93	0.26	0.93	0.26
Soccer network	Number of other communities with whom the community plays soccer[b]	5.99	2.54	6.01	2.52
Main channel (0/1)	Located along the main river (Amazon, Napo, Ucayali, Pastaza)	0.62	0.49	0.62	0.49
Floodplain soil (0-1)	Land underlain by Holocene parent material (proportion in 5km buffer land)	0.62	0.31	0.62	0.30
Forest (0-1)	Land classified to be forest in 2015 (proportion in 5km buffer land)	0.82	0.17	0.82	0.17
No. communities		466		433	

[a] One colonist community is treated as mestizo community. [b]Based on the original PARLAP community survey. [c]Based on 2007 population census.

Table S2. OLS estimates for number of all information sources

	Baseline Self-preventive behaviors (z-score) (1)	Hand-washing (0/1) (2)	Mask-wearing (0/1) (3)	Social distancing (z-score) (4)	Social restrictions (z-score) (5)	Follow-up Self-preventive behaviors (z-score) (6)	Hand-washing (0/1) (7)	Mask-wearing (0/1) (8)	Social distancing (z-score) (9)	Social restrictions (z-score) (10)
No. information sources	-0.0470*	0.0133	-0.00691	-0.0818***	0.0321	-0.00222	-0.00160	-0.0138	-0.0212	0.0293
	(0.0246)	(0.0123)	(0.0104)	(0.0310)	(0.0274)	(0.0161)	(0.0101)	(0.0110)	(0.0206)	(0.0200)
Indigenous (0/1)	0.0578	-0.102**	-0.00120	0.135	-0.0308	-0.0244	0.0155	-0.0679	0.0204	-0.0951
	(0.0857)	(0.0422)	(0.0384)	(0.100)	(0.0918)	(0.0618)	(0.0316)	(0.0472)	(0.0889)	(0.0735)
Population (log)	0.0272	0.0110	-0.0328	0.0532	0.0122	-0.0217	0.0235	-0.0633**	0.00679	-0.0447
	(0.0440)	(0.0236)	(0.0215)	(0.0598)	(0.0548)	(0.0348)	(0.0241)	(0.0277)	(0.0476)	(0.0376)
Distance from city (log km)	0.206***	0.0365	-0.0544	0.159*	0.205**	0.0891*	0.0125	0.0420	0.0827	0.0744
	(0.0757)	(0.0377)	(0.0431)	(0.0936)	(0.0988)	(0.0504)	(0.0321)	(0.0360)	(0.0709)	(0.0736)
Active public river transportation (0/1)	0.0601	-0.0867*	-0.0893	0.151	0.0128	-0.0549	-0.0555	-0.0110	0.0195	-0.0900
	(0.102)	(0.0513)	(0.0544)	(0.127)	(0.131)	(0.0943)	(0.0607)	(0.0574)	(0.116)	(0.0922)
Inactive public river transportation (0/1)	0.134	-0.109*	-0.0582	0.317*	-0.116	-0.199*	-0.0197	0.0279	-0.234*	-0.111
	(0.132)	(0.0600)	(0.0659)	(0.170)	(0.160)	(0.113)	(0.0790)	(0.0703)	(0.134)	(0.128)
Communication access (0/1)	0.129*	0.0545*	0.0347	0.0564	0.145*	0.121**	0.0216	0.110***	0.143**	0.0177
	(0.0688)	(0.0297)	(0.0304)	(0.0908)	(0.0773)	(0.0539)	(0.0336)	(0.0415)	(0.0719)	(0.0488)
Health facility (0/1)	0.0477	0.0813	0.0953*	0.127	-0.146	-0.0493	0.0412	-0.0955	0.0176	-0.115
	(0.112)	(0.0570)	(0.0496)	(0.133)	(0.136)	(0.0853)	(0.0605)	(0.0593)	(0.0931)	(0.0968)
Past epidemic (0/1)	-0.0158	0.0243	-0.0627	-0.115	0.0131	-0.00684	-0.00479	-0.00350	-0.0146	-0.00385
	(0.0787)	(0.0432)	(0.0414)	(0.109)	(0.0921)	(0.0662)	(0.0390)	(0.0496)	(0.0813)	(0.0697)
Past NGO activities (0/1)	0.0809	-0.00265	0.0484	0.137	0.0689	0.00676	-0.0154	-0.00360	0.0409	-0.0371
	(0.0717)	(0.0327)	(0.0339)	(0.0946)	(0.0741)	(0.0466)	(0.0317)	(0.0380)	(0.0656)	(0.0538)
Past health NGO activities (0/1)	0.0130	-0.0506	0.00513	0.00318	-0.0634	-0.0954	-0.0770	-0.0703	-0.172	0.122
	(0.111)	(0.0612)	(0.0549)	(0.156)	(0.125)	(0.101)	(0.0551)	(0.0662)	(0.132)	(0.117)

(continued)

	Baseline					Follow-up				
	Self-preventive behaviors	Hand-washing	Mask-wearing	Social distancing	Social restrictions	Self-preventive behaviors	Hand-washing	Mask-wearing	Social distancing	Social restrictions
	(z-score)	(0/1)	(0/1)	(z-score)	(z-score)	(z-score)	(0/1)	(0/1)	(z-score)	(z-score)
	(1)	(2)	(3)	(4)	(5)	(6)	(7)	(8)	(9)	(10)
Secondary school (0/1)	-0.0678	-0.103**	0.0242	0.00606	-0.220**	-0.105	-0.0829	0.0169	-0.0567	-0.0947
	(0.0909)	(0.0478)	(0.0390)	(0.115)	(0.107)	(0.0663)	(0.0512)	(0.0452)	(0.0728)	(0.0837)
Church (0/1)	-0.184*	0.0225	0.0775	-0.0660	-0.353***	0.00704	0.0679	0.144**	0.0844	-0.210**
	(0.0991)	(0.0525)	(0.0598)	(0.148)	(0.124)	(0.0878)	(0.0608)	(0.0585)	(0.112)	(0.0985)
Population nearby (log)	-0.0436	0.00951	-0.00326	-0.0780	0.00705	0.0370	0.0167	0.0787***	0.00435	0.0196
	(0.0393)	(0.0181)	(0.0173)	(0.0496)	(0.0494)	(0.0232)	(0.0199)	(0.0192)	(0.0309)	(0.0312)
Distance from nearest market town (log km)	-0.0120	-0.0152	-0.0121	0.0455	-0.0253	0.0256	0.00206	0.0108	0.0283	0.00939
	(0.0429)	(0.0227)	(0.0243)	(0.0546)	(0.0529)	(0.0329)	(0.0186)	(0.0228)	(0.0416)	(0.0381)
Distance from nearest community (km)	0.00361	-0.00169	-0.000581	0.00514	0.000108	-0.00312	0.000259	-0.000484	-0.00356	-0.00299
	(0.00268)	(0.00191)	(0.00180)	(0.00418)	(0.00417)	(0.00260)	(0.00186)	(0.00175)	(0.00301)	(0.00318)
Community market (0/1)	0.104	0.132*	-0.0298	-0.0409	0.00353	0.0105	-0.0875	-0.000362	0.0817	0.0699
	(0.111)	(0.0679)	(0.0712)	(0.177)	(0.157)	(0.0974)	(0.0815)	(0.0815)	(0.134)	(0.0692)
Soccer network	0.0161	0.0118*	-0.00556	0.0114	0.0335**	0.0135	-0.00438	-0.00748	0.0175	0.0155
	(0.0123)	(0.00668)	(0.00572)	(0.0164)	(0.0143)	(0.0109)	(0.00640)	(0.00800)	(0.0144)	(0.00981)
Main channel (0/1)	-0.0406	-0.00527	-0.0431	-0.108	0.0113	-0.0701	-0.0235	-0.00968	-0.0888	-0.0123
	(0.0700)	(0.0315)	(0.0347)	(0.0919)	(0.0827)	(0.0557)	(0.0327)	(0.0391)	(0.0685)	(0.0566)
Floodplain soil (0-1)	0.0515	0.0102	0.0751	0.0139	0.0436	0.0271	0.0175	-0.0239	0.0935	0.0237
	(0.134)	(0.0568)	(0.0659)	(0.167)	(0.155)	(0.113)	(0.0713)	(0.0883)	(0.135)	(0.120)
Forest (0-1)	-0.249	0.0850	0.0779	-0.573	0.0596	0.228	-0.118	0.207	-0.0270	0.539*
	(0.326)	(0.170)	(0.183)	(0.420)	(0.389)	(0.258)	(0.170)	(0.184)	(0.316)	(0.308)
Basin fixed effects	YES	YES	YES	YES	YES	YES	YES	YES	YES	YES
Interviewer fixed effects	YES	YES	YES	YES	YES	YES	YES	YES	YES	YES
N	420	458	461	447	445	429	432	432	432	429
R squared	0.722	0.680	0.681	0.484	0.600	0.807	0.554	0.591	0.624	0.786
Mean of dependent variable	0.125	0.607	0.536	0.239	-0.00485	-0.123	0.787	0.583	-0.248	0.00705

Robust standard errors are shown in parentheses. *p<0.1, **p<0.05, ***p<0.01

Table S3. Sample representativeness

	Baseline sample (0/1) (1)	Follow-up sample (0/1) (2)
Indigenous (0/1)	0.165***	0.140***
	(0.0366)	(0.0374)
Population (log)	0.0117	0.00955
	(0.0277)	(0.0275)
Distance from city (log km)	-0.0413	-0.0626
	(0.0380)	(0.0392)
Public river transportation access (0/1)	-0.0230	-0.0288
	(0.0399)	(0.0406)
Telephone access (0/1)	0.0821**	0.0375
	(0.0339)	(0.0341)
Health facility (0/1)	0.0329	0.0154
	(0.0557)	(0.0592)
Past epidemic (0/1)	0.0723**	0.0531
	(0.0365)	(0.0368)
Past NGO activities (0/1)	0.115***	0.119***
	(0.0341)	(0.0352)
Past health NGO activities (0/1)	-0.00912	-0.00769
	(0.0673)	(0.0683)
Secondary school (0/1)	0.0701	0.0896
	(0.0555)	(0.0574)
Population nearby (log)	0.0168	0.0178
	(0.0219)	(0.0217)
Distance from nearest market town (log km)	0.0484**	0.0515**
	(0.0205)	(0.0216)
Distance from nearest community (km)	-0.00311	-0.00247
	(0.00243)	(0.00233)
Community market (0/1)	0.0802*	0.0782*
	(0.0440)	(0.0444)
Soccer network	0.00304	0.00559
	(0.00704)	(0.00733)
Main channel (0/1)	-0.0479	-0.0443
	(0.0373)	(0.0389)
Floodplain soil (0-1)	0.0402	0.0376
	(0.0617)	(0.0635)
Forest (0-1)	-0.189	-0.174
	(0.172)	(0.173)
Basin fixed effects	YES	YES
N	919	919
R squared	0.244	0.211
Mean of dependent variable	0.510	0.473

Robust standard errors are shown in parentheses. *p<0.1, **p<0.05, ***p<0.01
